# Application Intensity and Spatial Distribution of Three Major Herbicides from Agricultural and Nonagricultural Practices in the Central Plain of Thailand

**DOI:** 10.3390/ijerph18063046

**Published:** 2021-03-16

**Authors:** Suphaphat Kwonpongsagoon, Chanokwan Katasila, Pornpimol Kongtip, Susan Woskie

**Affiliations:** 1 Department of Sanitary Engineering, Faculty of Public Health, Mahidol University, 420/1 Rajvithi Road, Bangkok 10400, Thailand; chanokwan.k@gmail.com; 2 Center of Excellence on Environmental Health and Toxicology (EHT), Bangkok 10400, Thailand; pornpimol.kon@mahidol.ac.th; 3 Department of Occupational Health and Safety, Faculty of Public Health, Mahidol University, 420/1 Rajvithi Road, Bangkok 10400, Thailand; 4 Department of Public Health, Zuckerberg College of Health Sciences, University of Massachusetts Lowell, One University Ave, Lowell, MA 01854, USA; Susan_Woskie@uml.edu

**Keywords:** herbicide, glyphosate, paraquat, 2,4-D, agriculture, non-agriculture, environmental contamination, health risk, Thailand

## Abstract

The herbicides glyphosate, paraquat, and 2,4-D play a significant role in Thailand. This paper is among the first study to describe the intensity of herbicide application and illustrate how the herbicides are extensively distributed over a large area through both agricultural and nonagricultural practices. Using a quick, economical, and simplified method of Material Flow Analysis together with spatial analysis, better data for the analysis of possible environmental herbicide contamination, human exposure, and related health risks for the general public and applicators can be developed. The findings from this study showed that in the study province, about 2.2 million kg of the active ingredients from the three targeted herbicides is applied annually. Pathway flow modeling with spatial analysis identified several local hotspots of concern based on the type of herbicide and crop/activity where it was used. Cassava planting was found to have the highest herbicide application activity, whereas rice cultivation was the major contributor of total herbicide mass, due to the wide area of cultivation in the province. The herbicide most likely to be applied at rates higher than recommended was 2,4-D, particularly on cassava and sugarcane farms.

## 1. Introduction

Intensive and extensive herbicide application can be a serious threat to ecosystems by altering their ecological quality. In Thailand, over the past decades, herbicides have played a significant role in intensifying crop production. According to national statistics, about 80% of annual pesticide imports are herbicides, with the remaining 20% comprising insecticides, fungicides, rodenticides, molluscicides, and growth hormones [1]. While herbicide consumption has consistently increased over time, fungicide and insecticide consumption has slightly decreased. Based on herbicide imports and national cropland area [1,2], the application of herbicides has increased about two folds from 2005 to 2018 (average 1.3 to 2.9 kg active ingredient (a.i.) per one ha of cropland). The top three herbicide imports to Thailand are glyphosate (glyphosate isopropyl ammonium), paraquat (paraquat dichloride), and 2,4-D (mainly 2,4-D dimethyl ammonium), ranging from 40–50%, 15–20%, and 11–18%, respectively [1]. These three chemicals alone account for 79% of all herbicide imports.

Globally, glyphosate, paraquat, and 2,4-D herbicides are the most well-known and widely used weed killers for protecting crops. Both glyphosate and paraquat are nonselective herbicides, used to control all types of weeds, whereas 2,4-D herbicide is a selective herbicide; it was formulated to kill only broadleaf weeds. Ideally, herbicide application should only affect the target weeds, but it also influences the surrounding environmental compartments, crops, and humans. As reported from numerous studies, these herbicide residues are detectable across media such as in soil, surface water, groundwater, food, and even humans in Thailand, as well as in other countries [3,4,5,6,7,8,9,10,11,12,13]. These findings indicate offsite transport, leaching, potential human exposure, and possible health risks. Monitoring environmental samples for pesticides is critically important to protect crops, ecosystems, and human health. However, time and budget constraints are major obstacles, especially when covering a large geographic region and multiple media. So far, little attention has been paid to the practical understanding of all actual post-application pathways leading to environmental exposures. Better understanding is needed on how pathways are impacted by the type of application processes/activities (sources), the methods of transport and transformation in the environment (pathways) that lead to residues in the different types of media, and the degree to which residues result in ecological and human exposure and health risk. One approach that can be used for this purpose is Material Flow Analysis (MFA), which uses mathematical modeling as a technique to investigate sources, pathways, and residues of pollutants. The procedure has been used to model sources as well as mass and substance flows through a system [14,15,16,17,18,19,20]. The procedure has been described in detail in Schaffner et al. [14,15,18].

Regarding the toxic effects of these three herbicides, glyphosate has low acute toxicity among mammals (group III—slightly hazardous substance), with a rat lethal dose (LD_50_) of 4230 mg/kg according to the hazard classification of pesticides recommended by the World Health Organization (WHO) [21]. Paraquat and 2,4-D have been classified as moderately hazardous substances (group II of the WHO classification), with LD_50_ ranging from 375 to 1200 mg/kg, and 150 mg/kg in rats, respectively [21,22,23]. Due to these LD_50_ reports, glyphosate and 2,4-D herbicides have less acute toxicity than paraquat. However, in terms of chronic toxicity as determined by the International Agency for Research on Cancer (IARC), both glyphosate and 2,4-D are classified as probable (group 2A) and possible (group 2B) human carcinogens, respectively [24,25,26]. In addition to cancers, both glyphosate and 2,4-D have been identified as endocrine disruptors, due to their effects on the thyroid and gonads [27,28,29,30,31]. They have also been identified as causing birth defects among humans [32].

Up until the present, actual pesticide use data has been neither documented nor publicly available for crop, farm, or relevant activities in Thailand. The few studies published concerning agricultural pesticide applications in Thailand have been limited in location and pesticide type. Most have focused on the northern highland areas, where insecticides were mainly used in vegetable farms, or else focused on a specific small-scale farming system, (i.e., rice paddies) [33,34,35]. In contrast, this study constitutes the first assessment of the whole picture of major herbicide use for cultivating economic crops and nonagricultural activities of interest throughout a whole province in the central plain of Thailand. Thus, this study aimed (i) to conduct a comprehensive survey of major herbicides used for both agricultural and nonagricultural practices in one Thai province in order to develop an understanding of herbicide loads on the environment and (ii) to produce a preliminary screening map of major herbicide sources in the study province to be used to target assessments of environmental contamination and health risk. To achieve the established objectives, the approach of Material Flow Analysis (MFA) combined with spatial analysis was employed.

## 2. Study Areas: Nakhon Sawan Province, Central Thailand

**Figure 1:**
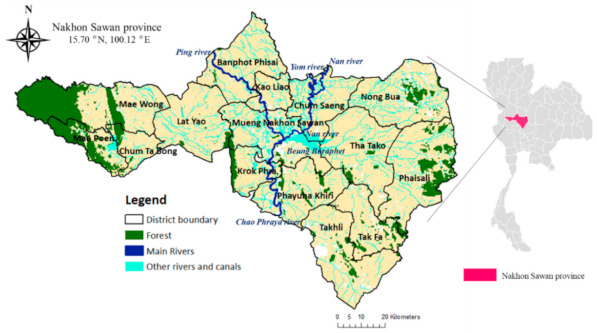
Boundary of Nakhon Sawan province, Thailand, showing boundaries of districts, forest, main rivers, and their tributaries.

Figure 1 shows the boundary of Nakhon Sawan Province, located on the central alluvial plain of Thailand, covering a total area of approximately 960,000 ha (or 9600 km^2^) [36]. The topography is mainly flat, transitioning to foothills and mountainous areas on the eastern and western borders. Geographically, the province is divided in 15 districts as shown in Figure 1, comprising a total of 130 subdistricts and 1328 villages. Three main rivers, the Ping, Yom, and Nan rivers, flow through the north of the province, then merge and form the Chao Phraya River at Muang Nakhon Sawan District (see Figure 1). Many tributaries of these rivers are interconnected by canals that serve for irrigating agricultural fields. The area is tropical savanna with an average annual temperature ranging from 20 to 34 degree Celsius. Due to the southwest monsoon blowing from the Indian Ocean, the rainy season period runs for six months, starting from May to October, with an average annual rainfall of 1150 mm [37].

Approximately 73% of the Nakhon Sawan area is agricultural land, followed by 10% water bodies, 9% forest, and 8% residential and industrial areas [38]. This proportion explains why Nakhon Sawan is ranked as one of the main agricultural production areas in Thailand. Rice is the major crop grown in the province, comprising about one half of the total cultivated area. Other major crop cultivation areas include sugarcane (17%), cassava (9%), and maize (5%) [38]. Other minor crops grown in the province include rubber tree, banana, mung bean, sesame, mango, guava, sweet corn, and some vegetables such as lime, cucumber, kaffir lime, cilantro, and chilies. According to the Thai Royal Irrigation Department [39], the cropping systems of Nakhon Sawan involve 111,000 hectares of irrigated areas, accounting for 16% of the total agricultural area. As a result of land use, the agricultural sector dominates the provincial economic structure, with about 45% of the population engaged in agricultural practices [40]. The agricultural economy in the province is comprised of sugarcane (59%), rice (20%), cassava (16%), and maize (1%) [38], corresponding to the national statistics.

In 2016, the total population of Nakhon Sawan was about 1,066,000 million, residing in 401,432 households situated across the province [40]. The average population density is about 110 people per 1 km^2^, but the densest distribution is along the main rivers and their tributaries. According to the Office of the Basic Educational Commission, the Ministry of Education [41], 555 schools, under the basic educational system, were located in the 15 districts of the province.

## 3. Materials and Methods

### 3.1. Material Flow Analysis Combined with Spatial Analysis

**Figure 2:**
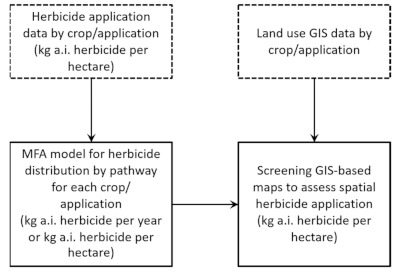
Schematic flow diagram for combining material flow analysis and land use analysis in this study.

Material Flow Analysis (MFA) and spatial analysis were combined as the method used in this study. The approach followed the method described in detail in the study of Kupkanchanakul et al. [42] for nutrient management in the river basin. Figure 2 shows the schematic flow diagram for combining MFA and land use analysis.

MFA was used to assess and provide a systematic description of mass and substance flows within a system defined in space and time, based on a mass balance principal. In general, the classical procedure of MFA involves system analysis, data collection, model equations, illustration of the results, and interpretation. More details can be found in related studies [14,15,16,17]. The MFA model can quantify sources, pathways, and discharge of substances such as nutrients (nitrogen and phosphorus) in a catchment or a city [14,15,18,19,42], or heavy metals in a country [17,20]. In the case of pesticides, the method has been used to track pesticide flows from application activities and to assess human exposures in a flower production greenhouse in Columbia [43].

**Figure 3:**
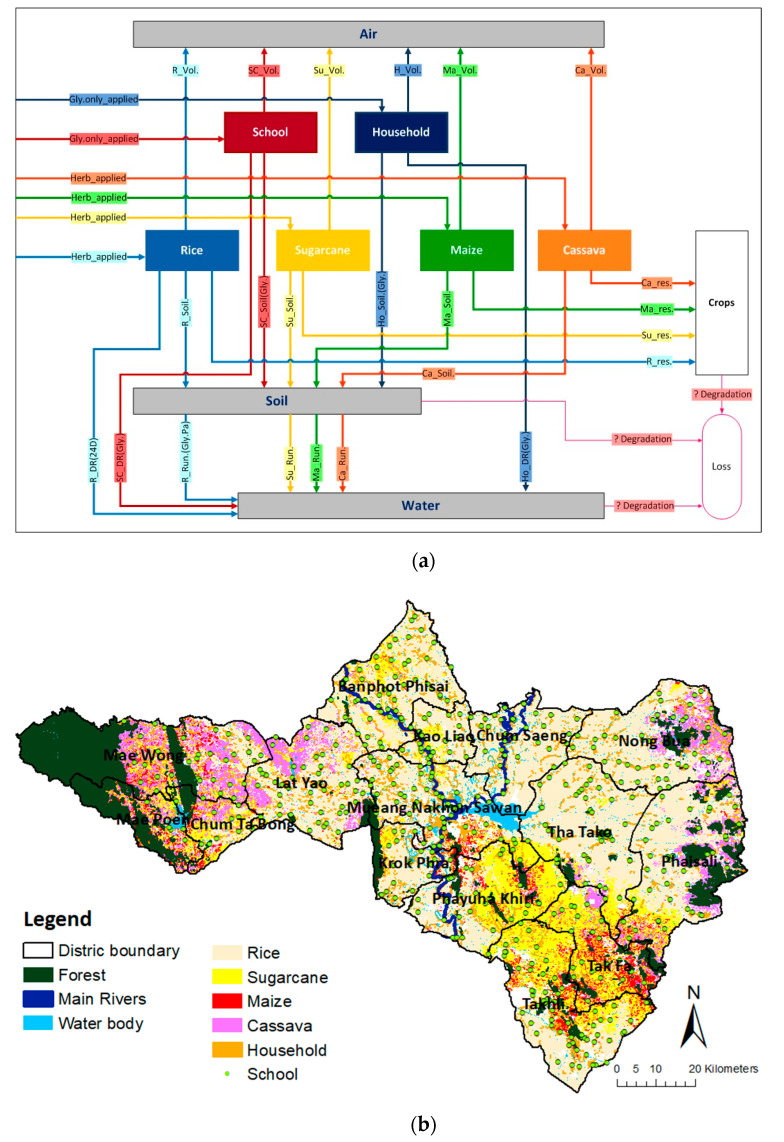
System analysis of herbicides (glyphosate, paraquat, 2,4-D) in Nakhon Sawan: (**a**) herbicide flow model by MFA; (**b**) spatial distribution of land use according to the MFA model. The abbreviation used in Figure 3a: rice (R), sugarcane (Su), maize (Ma), cassava (Ca), school (Sc), household (Ho), glyphosate (Gly), herbicides (Herb), volatilization from soil (Vol), runoff (Run), drainage (DR), and residue (Res).

For the MFA in this study, the spatial boundary of the system was defined as the province of Nakhon Sawan and the temporal boundary was the year 2016. The relevant application activities for the three target herbicides—glyphosate, paraquat, and 2,4-D were investigated in the study area of Nakhon Sawan. The application spraying activity targets included (1) rice, (2) sugarcane, (3) maize, (4) cassava, (5) school grounds, and (6) household grounds. The simplified MFA model is presented in Figure 3a. As seen in the figure, herbicides are distributed in the study area through those six spraying activity targets, and are transported to the surrounding environmental compartments (air, soil, and water) via volatilization from soil, drainage, and runoff. Herbicide residues can then be found in crops. However, herbicide loss by degradation processes also occurs during the application of herbicides in all environmental media. Within the system, herbicides flow through the six application activities, and their pathway flows were quantified in the unit of kilograms of herbicide active ingredient (a.i.) per year.

For the land use map, corresponding to the MFA model of Figure 3a mentioned above, a geographic information system (GIS) was applied to process spatial data representing various land use types and later to assess how the study areas was possibly contaminated with herbicide substances. The three types of data used for creating the background land use map included (1) administrative areas (boundaries) of the province and all districts; (2) land use database for 2016, including economic crops and residential areas; and (3) Global Positioning System (GPS) latitude and longitude coordinates of the educational institutes, which were obtained from Royal Thai Survey Department [44], Land Development Department (LDD) [45], and Office of the Basic Education Commission (OBEC) [46], respectively. Based on the GIS framework, the spatial distribution of land use corresponding to the model in Figure 3a was created as shown in Figure 3b, and ready for further use as a background map for subsequent analyses of herbicide use or load in this study.

### 3.2. Data Collection and Calculation

To obtain the actual amount of herbicide applied and relevant application details, e.g., herbicide product name, type, and formulation, active ingredient (a.i.), spraying method and equipment, number of crops yearly, size of school, and house lawns, etc., in-depth individual interviews were conducted intensively and applied to farm, house, and school levels in this study. All detailed information was gathered from focus participant groups associated with the herbicide use activities for both agricultural economic crop growers (rice, sugarcane, cassava, and maize), and for non-agricultural activities (home and school) through local people and staff of educational institutes in Nakhon Sawan. The interviews were conducted by random visits using the connections and participation of subdistrict leaders, village leaders, local people, and school teachers/staff in each district. All appointments were made in advance before conducting face-to-face interviews. The interview was carried out on a one by one basis, taking around 45 to 60 min for each person. Additionally, the surrounding area of pesticide storage and disposal and crop fields were observed wherever applicable. During the interviews, all prepared questions and follow-up questions based on interviewee’s responses were simply asked. The recorded data about herbicide products were immediately checked to confirm the accuracy by cross-checking with the secondary data on herbicide name, type, formulation, and percentage of a.i. actually available in the local market.

The interviews were conducted from November 2016 to July 2018. Because many farmers grow more than one type of crop yearly, a total of 110 datasets were collected from 61 interviewees (58 crop growers and local residents, and three educational institute’s workers). These datasets covered usage for 36 rice paddies, 19 sugarcane farmlands, 13 maize farmlands, 22 cassava farmlands, 17 residential areas, and three schools. The interviewees resided in 10 of the 15 districts in Nakhon Sawan (Mueng Nakhon Sawan, Nong Bua, Banphot Phisai, Kao Liao, Takhli, Tha Tako, Phayuha Khiri, Lat Yao, Mae Wong, and Tak Fha).

In addition to the herbicide application data collected above, the next group of input data required in this study included crop planting, school, and household areas [38,41,44,45,46]. Moreover, the last input data gathered for this study were transfer coefficients, i.e., the fraction of herbicide transferred from the application processes to the environment. The specific transfer coefficients were determined from the physicochemical properties of the three herbicides, literature data, and knowledge of herbicide practices in the studied area. A list of these specific transfer coefficients used for the herbicide flow model in this study is depicted in Table 1.

After collecting data described above, the herbicide application rate was firstly calculated for each dataset by multiplying the amount of herbicide use per hectare with the percentage of product a.i. in relation to each application activity, i.e., each type of crop planting, home use and school use. Based on the total area of crop planting/application, the total herbicide mass flow associated with the application activities (Figure 2) could be individually estimated in kilograms or tonnes a.i per year as expressed in Equation (1) below. All three herbicide uses, overall economic crops, and other related activities are summed to exhibit the total provincial amount of herbicides applied per land use area.

(1) $$M_{tot}=\left[\frac{\Sigma \left(\frac{Mp}{a}.Hai\right)}{N}\right]\times A$$

where *M_tot_* denotes total amount of herbicide active ingredient (a.i.) application (kilogram a.i per year). *M_p_* denotes amount of herbicide product applied for one dataset (kilogram). *a* is total area applied for one dataset (hectare). *H_ai_* is percentage of active ingredient in herbicide product (%). *A* and *N* denote total areas for each crop planting/application area (hectare) and number of interview datasets, respectively.

As mentioned above, the MFA herbicide flow model (Figure 3a) was then used, showing the kilograms or tonnes of herbicide per year for each type of application/activity, and these herbicide loads were then normalized to the reference area for each activity, providing specific herbicide loads in kilograms a.i. herbicide per hectare per year. Using the prepared land use map together with specific load estimations of the MFA model, a spatial distribution for herbicide loads was produced (see Figure 2).

## 4. Results

### 4.1. Major Herbicide Uses

The in-depth interviews provided the opportunity to obtain a better understanding of how pesticides are used relative to activities/crops. It provides not only information on the three target herbicides, but also other pesticides applied in the province. The results revealed that 15 types of pesticides were commonly used for different activities in the study areas, namely, glyphosate, paraquat, 2,4-D, acetochlor, alachlor, ametryn, atrazine, bispyricbac-sodium, butachlor, diuron, pendimenthalin, pretilachlor, abamectin, chlorpyrifos, and cypermethrin. This indicated that 80% of pesticide types used in the area were identified as herbicides, with the remaining insecticides totaling 20%. In agriculture areas, rice cultivation alone applied about nine different kinds of herbicides (glyphosate, paraquat, 2,4-D, acetochlor, ametryn, bispyricbac-sodium, butachlor, pendimenthalin, and pretilachlor), whereas sugarcane, cassava, and maize plantations consumed about seven different herbicide types for each crop. For nonagricultural areas, both glyphosate and paraquat were mentioned to control and kill any unwanted weeds in school lawns and surrounding outdoor areas of the interviewees’ houses, but only glyphosate use was reported quantitatively during the period of data collection. Among the herbicides, glyphosate and paraquat were the most commonly used for both agricultural and nonagricultural activities.

The three target herbicides are available in a variety of trade and brand names. Concerning the formulations, only glyphosate 48% in soluble liquid concentrate (SL) (or glyphosate isopropyl ammonium (48%) and paraquat 27.6% SL (or paraquat dichloride 27.6%) were used in the study areas. In the case of 2,4-D, the two most common formulations included 2,4-D-dimethyl ammonium salt 84% SL and 2,4-D sodium salt 95% in water soluble power (SP), as reflected in Thai import statistics. The use of 2,4-D-dimethyl ammonium salt 84% SL was much more popular among farmers than the use of 2,4-D sodium salt 95% SP, accounting for 78% of the usage. This may be because of its liquid form, good water solubility, and ease of mixing before applying as sprays. In addition, 2,4-D dimethyl ammonium salt 84% SL was applied more often on rice and sugarcane crops, whereas 2,4-D sodium salt 95% SP was favored for cassava.

Regarding application equipment, two common types of sprayers were used in the study areas, namely, a single nozzle knapsack sprayer with a 20 or 25 L tank, and a tractor mounted with multi-nozzle sprayer with a 200 or 1000 L tank. In most cases, the farmers preferred the knapsack sprayer, while the multi-nozzle sprayer was more likely to be used in a large-scale planting area with large gaps between crop rows, like sugarcane fields. For the spray application of the three target herbicides, the growers typically sprayed herbicides directly onto soils and weeds in the treated areas, around the base of cassava plants, on soil on the paddy-field ridge, and between sugarcane and maize planting rows. In contrast, a broadcast spray application usually involved a selective herbicide, e.g., 2,4-D, commonly used to treat weeds in an entire area such as rice paddies and sugarcane fields.

**Figure 4:**
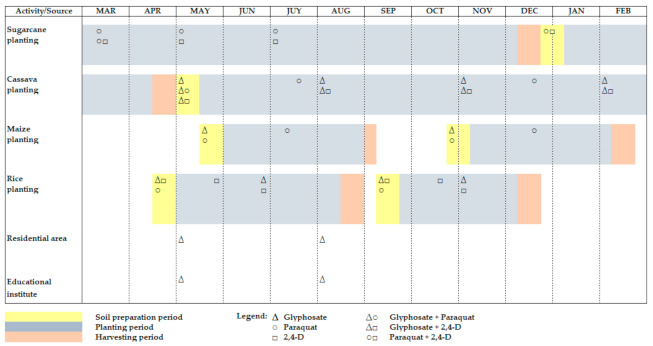
Period of herbicides application in Nakhon Sawan, central plain of Thailand.

Figure 4 presents the period of herbicide application for six different activities/crops of interest in the study areas of central Thailand. Clearly, herbicides glyphosate, paraquat, and 2,4-D are spread over the land many times throughout the year. In the agricultural areas, these three herbicides were used during soil preparation and crop planting periods. Considering all agricultural and nonagricultural activities, the herbicides were most frequently used (about 16 times) during wet season (May to October), compared with about eight times during dry season (November to April). Moreover, growers were likely to mix two or more herbicides a.i. in a single solution. Among the three target herbicides, 2,4-D was usually mixed with other herbicides, (2,4-D+glyphosate, 2,4-D+butachlor, and 2,4-D+ametryn+atrazine), because 2,4-D kills only broadleaf weeds, and farmers wanted to control more weeds simultaneously, saving time and labor for spraying. However, many just simply imitated the mixing recipes initiated by other neighboring growers. For example, glyphosate mixed with 2,4-D was applied in rice paddies and cassava fields, glyphosate combined with paraquat was used on cassava plantations, and a mixture of paraquat and 2,4-D was applied in sugarcane fields (see also Figure 4).

Table 2 presents the intensity of annual herbicide application in Nakhon Sawan, the central plain of Thailand. The results indicated that the application rate of herbicides differed significantly among activities and crops. Overall, the application rate for the three herbicides ranged from 0.96 to 7.83 kg a.i. per hectare for school use and cassava growing, respectively. Individual herbicide use indicated that the highest application rate for glyphosate was found in residential areas (4.39 kg a.i. per hectare), followed by 2,4-D used for sugarcane (2.44 kg a.i. per hectare) and paraquat in cassava fields (1.89 kg a.i. per hectare). Clearly, in terms of herbicide application rate, glyphosate and paraquat were applied most intensively in cassava fields. However, since the largest area under cultivation was rice (about 50% of total agricultural area) and rice has a high annual application frequency (see also Figure 4), rice farming contributed the most herbicide mass in the province.

Table 3 presents the percentage of users who applied herbicides areas above the recommended rates. According to weed science management and the recommendations for the use of herbicides [61], the recommended dose for 2,4-D is 1 kg a.i. per hectare. This study revealed that about 60% of all farmers applied 2,4-D at rates above this, especially when applying in sugarcane and cassava fields. Almost 80% of cassava growers used excess paraquat (over 1 kg a.i. per hectare), but none of the maize growers applied paraquat excessively in their planting fields. For glyphosate, all farmers and local users in rice, maize, and school areas, applied an amount lower than the recommended dose of 3 kg a.i. per hectare.

### 4.2. Current State of Herbicide Flows

**Figure 5:**
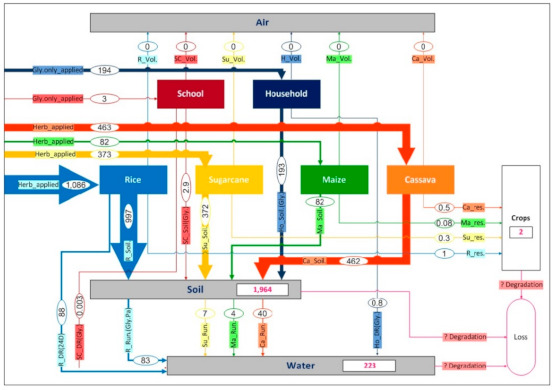
Herbicide flow model for glyphosate, paraquat, and 2,4-D applications in Nakhon Sawan Province (tonne a.i. per year).

Figure 5 shows the current state of the herbicide pathway flows from the model for Nakhon Sawan in 2016. The model provides a good overview of all relevant herbicide flows presented in tonnes per year, with a total loading of around 2.2 million kg a.i. per year, resulting from six herbicide application activities in the province (see also Table 2). The total herbicide application loadings estimated for soil and water are of particular interest.

As these herbicides were released directly into soil and on unwanted weeds in the treated areas, all uses of the target herbicides (2.2 million kg a.i. per year) were identified as producing mass loading on the soil/land. Moreover, about 10% of the total herbicide mass applied (or around 223 tonnes a.i. per year) was flowed to the receiving water bodies of the study area, resulting in mass loading there. Rice paddy fields were the major contributors of 2,4-D via water drainage (39% or 88 tonnes a.i. per year) due to the water management practices and glyphosate and paraquat via surface runoff (37% or 88 tonnes a.i. per year) due to rainfall (see Figure 5).

### 4.3. Screening GIS-Based Maps

**Figure 6:**
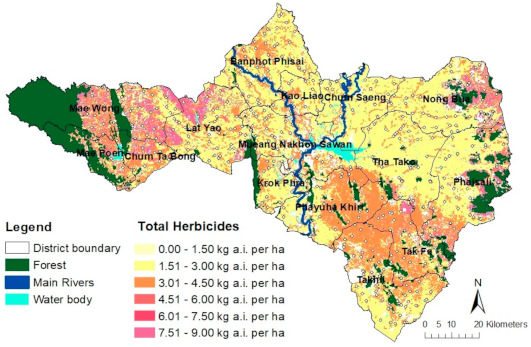
Spatial distribution of total herbicide loading (kg a.i./ha/yr) through various application sources in Nakhon Sawan Province, Thailand.

Using the estimates of annual average application rates and specific herbicide loads for all three target herbicides in kilogram herbicide a.i. per hectare per year, the data were then manipulated, analyzed, and spatially distributed in the form of a GIS-based screening map (spatial distribution) as shown in Figure 6. Figure 6 shows a spatial pattern indicating the highest values (hotspots) for the combined herbicides (7.51 to 9.00 kg a.i. per ha, shown in purple) applied in western (Mae Wong, Mae Poen, and Chum Tabong Districts) and eastern areas (Nong Bua and Phaisali Districts), where cassava tended to be more abundant, adjacent to forest (see Figure 3b for land use map). In addition, the higher herbicide application ranging from 3.01 to 4.50 kg a.i. per ha shown in orange in the southern part and along the receiving water bodies of the province where sugarcane fields and residential areas are situated, respectively. Widespread areas of the province growing rice and maize indicated in yellow received herbicide loads in a range from 1.50 to 3.00 kg a.i. per ha per year. Moreover, the lightest yellow spots dispersed throughout the study area represent the lowest herbicide application rates in school areas.

**Figure 7:**
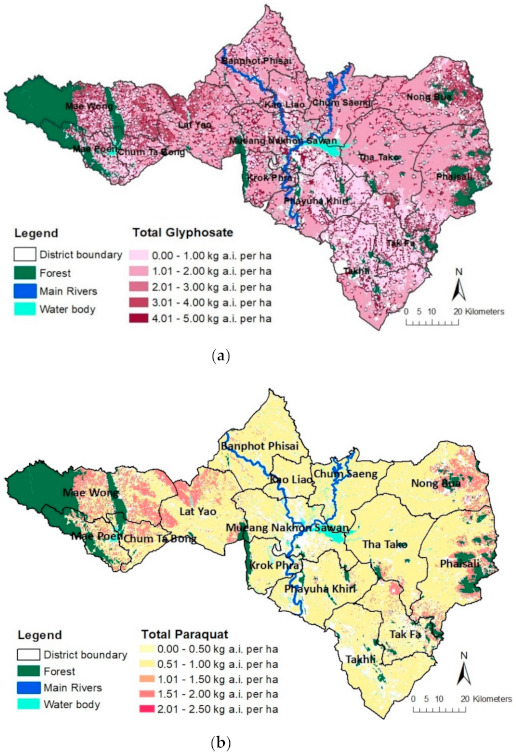

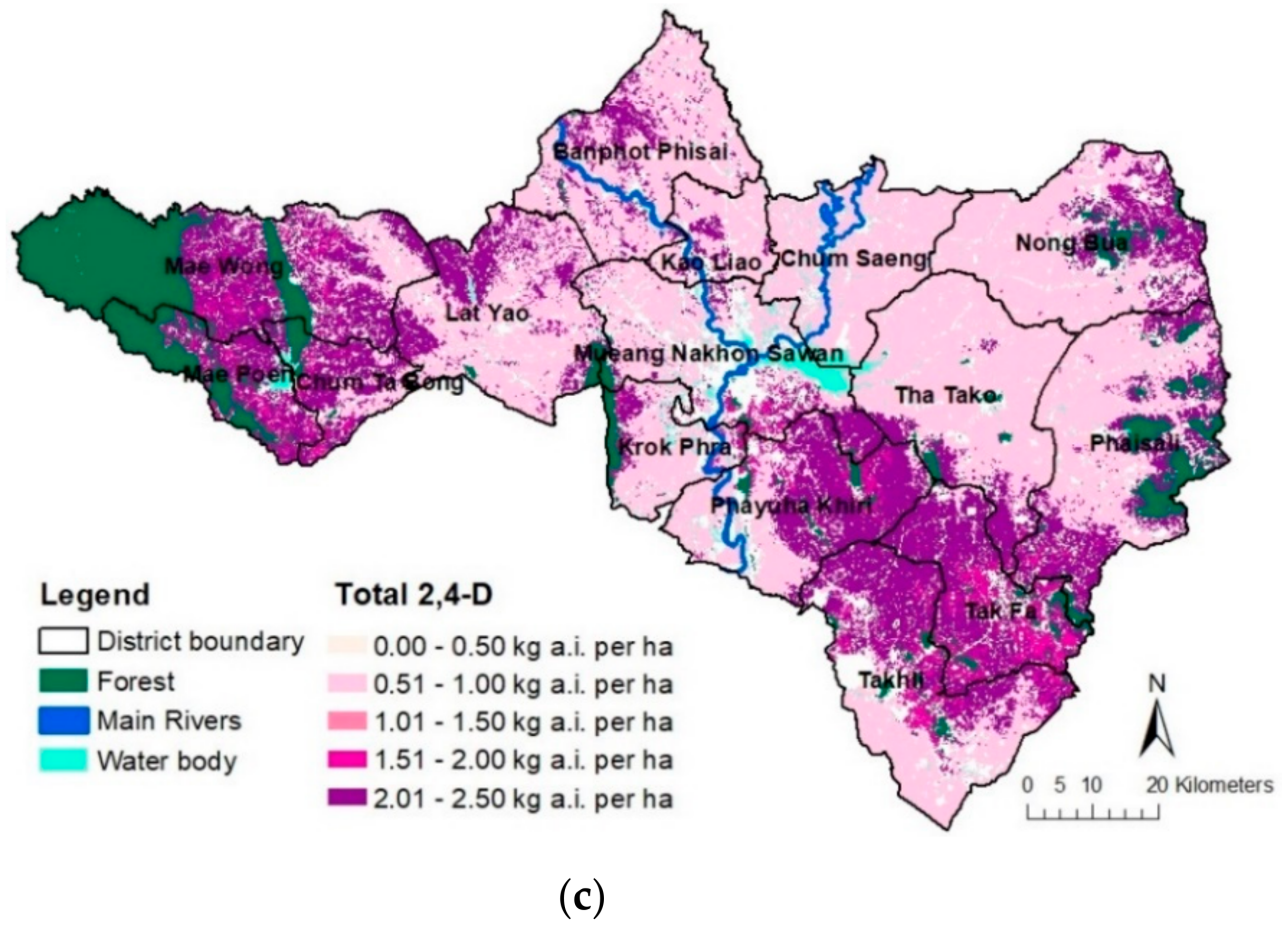
Spatial distribution of individual herbicide loading (kg a.i./ha/yr) from various application sources to Nakhon Sawan Province, Thailand: (**a**) glyphosate; (**b**) paraquat; (**c**) 2,4-D.

When considering the herbicides separately (glyphosate, paraquat, and 2,4-D), each herbicide loading to the study area could also be spatially distributed as displayed in the GIS-based maps in Figure 7a–c. Figure 7c highlights the potential hot spot areas of herbicide 2,4-D in western (Mae Wong, Mae Poen, and Chum Tabong Districts), southern (Phayuha Khiri, Tak Fa, and Takhli Districts), and eastern areas close to the forests and hills (Nong Bua and Phaisali Districts), where cassava and sugarcane dominate. For glyphosate loading to the land area, Figure 7a indicates the hot spots (the darkest and the second darkest purple) scattered throughout Nakhon Sawan Province, resulting from outdoor household use and cassava planting. Paraquat was highlighted in cassava planting fields in western and eastern areas near the forests and border of the province (see Figure 7b).

## 5. Discussion

When the application rate of pesticides in this study is compared to other studies, we see that the application of 2,4-D on sugarcane crops was approximately 10-fold higher in Thailand than that reported in Brazil, the world’s largest sugarcane producer [62] (Table 4). For the herbicides paraquat and glyphosate, their intensive uses in cassava fields is about 4.4 and 2.8 times higher, respectively, than in the cassava fields of Ireland. For rice and maize areas in Thailand, the use of all herbicides were within the same range reported in related studies, except that Sri Lanka reported 3.7 times higher use of glyphosate for rice paddies. Some of these overuses may be attributable to the high frequency with which Thai farmers mix herbicide cocktails when spraying their fields (Figure 4). Mixing pesticide cocktails has also been reported in other Asian countries, e.g., India, Sri Lanka, and Cambodia [63,64,65,66,67]. Looking at the nonagricultural sector, use of glyphosate in residential areas in Thailand was much higher than in the UK and in Belgium, even though Belgian residential use represented a worse-case scenario (unusual case) [56].

One reason for higher application rates in Thailand is the tendency of Thai farmers/users to utilize greater than recommended application rates, as described previously in the results Section 4.1 (see also Table 2). In this study, we report that 19% of glyphosate users, 35% of paraquat users, and 61% of 2,4-D users over applied these herbicides (Table 3). In cassava and sugarcane fields, this was particularly true. Similar reports of pesticide overuse were reported in the intensive upland vegetable production system in northern Thailand [33], as well as in other studies [65,68,69,70].

In comparing pesticide application rates, other factors in addition to the farmer’s behavior impact the intensity of herbicide application in different regions, especially European countries. Specific variations in climatic factors such as rainfall, air temperature, humidity, and soil characteristics, which can influence plant growth and the length of the vegetation period, will all impact the application rates and frequencies of pesticide usage.

The MFA model of herbicide flow developed here demonstrated that the entire province received a massive annual load of the three herbicides (glyphosate, paraquat, and 2,4-D) estimated at about 2.2 million kg a.i.per year. The model focused on soil and water as the major pathway flows for these pesticides. Due to the physiochemical properties of herbicides together with the basic spraying methods practiced in the study area (backpack spray pointed at the ground), all herbicides were chiefly deposited directly onto the soil, and spray drift offsite was not incorporated into the model. With the relatively low vapor pressure values of these herbicides, they all exhibited a very low tendency to volatilize in air, particularly under conditions of high relative humidity [79,80]. The model identified soil as the major pathway for herbicide loading. Among the three target herbicides, both glyphosate and paraquat had high soil sorption coefficients (Koc), so were very strongly adsorbed by soil particles, leading to very low mobility [81]. On the other hand, 2,4-D showed a lower tendency to bind to soil because of its high-water solubility and lower Koc value [81]. The flow results from the model were consistent with the study of pesticide use and residual occurrence in soil and water in northern and eastern Thailand reported by Thapinta and Hudak [82], indicating the levels of pesticide concentration detected in soil were much higher than those in water, confirming a tendency for pesticides to be absorbed in soil particles in the area.

The model developed does contain several limitations. One improvement of the model would be to include herbicide loss by degradation processes in soil, water, and crops as another transformation pathway. In addition, the model estimates an average level of herbicide load in each pathway for each herbicide and crop/application; thus, the mapping does not provide data on the variability in loading at the level of a single farm, house, or school; rather, it is assumed that loadings are uniform across each type of crop/application. We were unable to include all possible sources of herbicide use; for example, we did not collect data on the use of herbicides in railway and roadside applications.

In line with the related study of nutrient management for the river basin by Kupkanchanakul et al. [42], our findings confirmed that using the MFA approach, combined with spatial analysis in the form of screening GIS-based maps, could identify where residues are likely to be detected in soil and nearby river tributaries, leading to widespread environmental contamination. All findings obtained from the simplified herbicide flow model and GIS-based screening maps could be used directly as a basis for a water and soil sampling campaign as well as for discussion among stakeholders, e.g., private, local government, farmers, local people, educational institutes, etc., on possible reduction or restriction measures to the use of herbicides and the potential adverse impacts of herbicide use for the local environment and health of both applicators and general public. In addition, this type of modeling could be linked with location based health data to assess health risk [83,84,85,86].

A significant advantage of the MFA/spatial analysis method is the relative cost-effectiveness compared to widespread field measurements. The implementation of a program of widespread field measurements of soil and water for the large number of pesticides used in a developing country, like Thailand, is difficult due to financial and personnel limitations within the assigned government regulatory bureaus. However, the herbicide flow model was simply developed, and although estimates were based on some broad assumptions and approximations, it provides a starting point for future investigations and interventions.

## 6. Conclusions

The present study found that using the approach of MFA combined with spatial analysis, herbicide application intensity and its spatial distribution from a wide range of activities could be implemented across a large-scale area (province). In this study, the three major herbicides (glyphosate, paraquat, and 2,4-D) were applied intensively over the province around 2200 tonnes a.i. annually. The average total application rate ranged from 0.96 kg a.i. per hectare per year used in school lawns to 7.83 kg a.i. per hectare per year applied on cassava fields. All three herbicides were distributed in the environment, mainly to soil. In terms of the total mass and extensive application, rice cultivation clearly presents a potential source of herbicide contamination to the environment, while cassava planting was the most significant agricultural activity contributing the highest herbicide loading to soil and nearby receiving water bodies. This method allowed the identification with GIS-based screening maps of hotspot areas of possible high-risk exposure to these three herbicides. This type of information could benefit all stakeholders, aiding in the development of reduction or restriction measures regarding the use of herbicides.

## Figures and Tables

**Table 1:** Specific transfer coefficients used to estimate herbicide flows in this study.

Environmental Transport Process	Parameter Name	Specific Transfer Coefficient			Reference
		Glyphosate	Paraquat	2,4-D	
Direct transfer into soil	R_Soil	0.99	0.99	0.75	[47,48]
	Su_Soil	N/A	0.99	0.99	
	Ma_Soil	0.99	0.99	0.99	
	Ca_Soil	0.99	0.99	0.99	
	Sc_Soil	0.99	0.99	0.99	
	H_Soil	0.99	0.99	0.99	
Overland flow to water: runoff	R_Run.	0.15 [49,50]	0.07 [51]	N/A	[49,50,51]
	Su_Run.	N/A	0.07 [51]	0.005 [52,53]	[51,52,53]
	Ma_Run.	0.15 [49,50]	0.07 [51]	0.005 [52,53]	[49,50,51,52,53]
	Ca_Run.	0.15 [49,50]	0.07 [51]	0.005 [52,53]	[49,50,51,52,53]
Direct transfer into water: drainage	R_DR	N/A	N/A	0.25	[54]
	Sc_DR	0.001	N/A	N/A	[55,56]
	H_DR	0.001	N/A	N/A	[55,56]
Direct transfer into air	R_Vol.	0	0	0	[53,57,58]
	Su_Vol.	N/A	0	0	
	Ma_Vol.	0	0	0	
	Ca_Vol.	0	0	0	
	Sc_Vol.	0	0	0	
	H_Vol.	0	0	0	
Uptake by crops	R_res.	0.001	0.001	0.001	[47,59,60]
	Su_res.	N/A	0.001	0.001	
	Ma_res.	0.001	0.001	0.001	
	Ca_res.	0.001	0.001	0.001	

**Table 2:** Intensity of major herbicides application in Nakhon Sawan during a one-year period.

Activity/ Source	Land Area	App. Freq. (time year^−^^1^)	All Herbicides			Glyphosate			Paraquat			2,4-D		
	(1000 ha)		App. Rate(kg a.i. ha^−^^1^)	Total Mass Applied(kg a.i yr^−^^1^)	Share (%)	App. Rate(kg a.i. ha^−^^1^)	Total Mass Applied (kg a.i yr^−^^1^)	Share (%)	App. Rate (kg a.i. ha^−^^1^)	Total Mass Applied (kg a.i yr^−^^1^)	Share (%)	App. Rate (kg a.i. ha^−^^1^)	Total Mass Applied (kg a.i yr^−^^1^)	Share (%)
Rice	376	6	2.89	1,086,318	49	1.08 ± 0.40	399,858	48	0.89 ± 0.38	333,662	61	0.92 ± 0.75	352,798	42
Sugarcane	117	4	3.16	373,008	17	n/a	n/a	n/a	0.72 ± 0.75	85,473	16	2.44 ± 1.38	287,535	35
Cassava	59	6	7.83	463,357	21	3.56 ± 2.00	210,911	25.5	1.89 ± 1.19	111,838	21	2.38 ± 1.25	140,607	17
Maize	28	4	2.85	82,396	4	0.63 ± 0.00	18,101	2	0.47 ± 0.25	13,614	2	1.75 ± 1.52	50,682	6
Household	44	2	4.39	193,776	8.9	4.39 ± 3.86	193,776	24	n/a	n/a	n/a	n/a	n/a	n/a
Schools	3	2	0.96	2969	0.1	0.96 ± 0.57	2,969	0.5	n/a	n/a	n/a	n/a	n/a	n/a
Sum	627	24	22**.**08	2,201,824	100	10**.**62	825,615	100	3**.**97	544,587	100	7**.**49	831,622	100

Note: App. = Application; Freq. = Frequency; n/a = not applicable.

**Table 3:** Percentage of herbicides used excessively by activities/areas.

Activity/Area	Excessive Use (% Growers or Users)		
	Glyphosate	Paraquat	2,4-D
Rice plantation	0	40	37
Sugarcane plantation	n/a	18	92
Cassava plantation	47	78	75
Maize plantation	0	0	50
Residential area	33	n/a	n/a
Educational institute area	0	n/a	n/a
Overall	19	35	61

**Table 4:** Comparison of herbicide application intensity with other studies.

Sector	Herbicide/Study Area	Crop/Source	Application Rate	Reference
			(kg a.i. ha^−1^)	
***Agriculture:***	***Glyphosate***			
	*Nakhon Sawan, Thailand*	*All crops ^a^*	1.92	*This study*
		*Rice*	1.08	*This study*
		*Cassava*	3.56	*This study*
		*Maize*	0.63	*This study*
	Mahaweli river basin, Sri Lanka	Rice Paddy	3.96 *	[71]
	The Cagayan Valley, Northern Philippines	Rice	0.47	[72]
	Ireland	Cassava	1.25	[73]
	Denmark	Maize	1.44	[50]
	Flanders, Belgium	Maize	0.25	[74]
	***Paraquat***			
	*Nakhon Sawan, Thailand*	*All crops ^b^*	1.04	*This study*
		*Rice*	0.89	*This study*
		*Sugarcane*	0.72	*This study*
		*Cassava*	1.89	*This study*
		*Maize*	0.47	*This study*
	India	Rice	0.53	[75]
		Maize	0.69	[75]
	Ireland	Cassava	0.43	[73]
	The State of Pernambuco, Brazil	Sugarcane	0.71	[76]
	***2,4-D***			
	*Nakhon Sawan, Thailand*	*All crops ^b^*	1.65	*This study*
		*Rice*	0.92	*This study*
		*Sugarcane*	2.44	*This study*
		*Cassava*	2.38	*This study*
		*Maize*	1.75	*This study*
	West Bengal, India	Rice	2.00	[77]
	The Cagayan Valley, Northern Philippines	Rice	0.03	[72]
	Laguna and Quezon Province, the Philippines	Rice	0.80	[78]
	The State of Pernambuco, Brazil	Sugarcane	0.25	[76]
***Non-agriculture:***	**Glyphosate**			
	*Nakhon Sawan, Thailand*	*Residential*	4.39	*This study*
	York, UK	Residential	0.15	[55]
	Meerhout, Belgium	Residential	3.71	[56]

^a^ rice, cassava, and maize. ^b^ rice, sugarcane, cassava, and maize. * estimated from 36% glyphosate use.
